# Static and dynamic mechanical properties of 3D-printed clear resin with embedded orthodontic metal wire

**DOI:** 10.1186/s40510-025-00559-1

**Published:** 2025-03-24

**Authors:** Junjing Zhang, Yuqiang Zhang, James Kit Hon Tsoi, Yanqi Yang, Kiho Cho

**Affiliations:** 1 Faculty of Dentistry, Dental Materials Science, Applied Oral Sciences & Community Dental Care, The University of Hong Kong, Hong Kong, SAR PR China; 2 Faculty of Dentistry, Division of Paediatric Dentistry and Orthodontics, The University of Hong Kong, Hong Kong, SAR PR China

**Keywords:** 3D-printed clear resin, Orthodontics, Mechanical property, Stress relaxation, Embedded metal wire, Multi-materials

## Abstract

**Background:**

The mechanical properties of directly 3D-printed clear dental aligners are currently constrained by the limitations of available 3D printing materials. This study aimed to investigate the mechanical properties of direct 3D-printed clear resin embedded with orthodontic wire under different surface treatments to propose a novel integration method for orthodontic appliances and treatment.

**Methods:**

The stainless-steel wires were divided into three groups based on surface treatments: control groups (C), sandblasting group (S), sandblasting and acid etching group (SA). Surface characteristics were analyzed and interfacial shear strength (IFSS) was measured. Dumbbell-shaped specimens were fabricated using 3D-printed clear resin and divided into four groups, depending on whether they were embedded with stainless-steel wires subjected to different surface treatments. The static and dynamic mechanical properties tests were carried out to calculate elastic modulus, tensile strength, and stress relaxation.

**Results:**

The average roughness and surface morphology of stainless-steel wires exhibited significant differences (*P* < 0.001) following different surface treatments. Sandblasting and acid-etching significantly enhanced IFSS, resulting in a fivefold increase to 28.8 MPa. The elastic modulus and tensile strength of the 3D-printed resin embedded with wires were significantly higher than those of the pure 3D-printed resin group. However, no significant differences in elastic modulus were observed among the different wire surface treatment groups. The sandblasting and acid-etching group exhibited higher residual stress compared to the other groups during both 6-hour and cyclic stress relaxation tests.

**Conclusion:**

This study presents a novel approach to 3D-printed clear dental aligners integrated with metal wires for orthodontic treatment. Surface treatment of orthodontic metal wire through sandblasting and acid etching enhances the bonding strength between the wire and 3D-printed clear resin, improving the static and dynamic mechanical properties of directly 3D-printed clear resin appliances. The innovative process and device provide an integrated solution for digital orthodontic treatments.

## Introduction

With the increasing demand for aesthetic and comfortable orthodontic solutions, clear aligner treatments have gained widespread popularity, particularly among adult patients [1, 2]. Although clear aligners have a wide range of applications and improved significantly, the mechanical properties of clear aligners remain a limiting factor for achieving complex tooth movements [3, 4]. Additionally, traditional thermoformed aligners are associated with time and cost inefficiencies and dimensional instability [5, 6].

The advancement of 3D printing technology will enable personalized and customized orthodontic treatment procedures [7, 8]. Directly 3D-printed orthodontic aligners have emerged as a promising alternative, providing better geometric accuracy, fit, and efficacy [1, 9, 10]. 3D-printed aligners eliminate the need for physical tooth models, streamline supply chains, and reduce time and costs.

Several studies have explored various aspects of directly 3D-printed orthodontic clear aligners [11], including printing accuracy, dimensional accuracy, and the effect of printing orientation [9, 12, 13]. Marco et al. [14] demonstrated that 3D-printed aligners could be utilized for mildly crowded cases with equivalent tooth movement accuracy. 3D printing allows precise shape and thickness control over specific appliances, which is challenging with conventional methods [15]. Moreover, 3D-printed resins showed promising shape memory properties, improving adaptability and reducing force decay [16, 17]. Although some studies have reported superior mechanical and physical properties of 3D-printed clear resin [18, 19], research on the mechanical performance of 3D-printed aligners remains limited. It was worth noting that Shirey et al. [20] found that 3D-printed aligners exhibit lower mechanical properties than thermoformed aligners, potentially compromising their ability to generate and retain tooth movement forces.

Clear aligners and retainers need to provide consistent and gentle forces during tooth movement, especially for long-term use, while maintaining sufficient mechanical strength to support complex orthodontic treatments. Therefore, high yield and ultimate tensile strength, appropriate elastic modulus, low stress relaxation rate, and biocompatibility are necessary [8, 21]. The multi-component systems that incorporate various materials and properties can be tailored to achieve specific orthodontic movements and desired mechanical characteristics [22]. The mechanical performance of 3D-printed polymers can be enhanced by incorporating reinforcing materials, such as metal wires, into the resin matrix [23, 24]. The literature reported that metal-wire reinforced composite bridges have improved restoration survival rates [25], and a relapsed anterior open bite case was treated with newly designed modified 3D-printed aligners with biting springs [11]. Hybrid therapies combining clear aligners with partial fixed lingual appliances have also proven effective for cases requiring high orthodontic forces or unpredictable tooth movements [26]. Stainless steel orthodontic wires are widely used due to their corrosion resistance and mechanical properties. Surface treatments such as sandblasting, chemical etching, and the application of metal bonding agents have been shown to enhance the interfacial bonding strength between wires and resins [27]. The integration of metal-polymer multi-materials is increasingly being adopted across various industries, highlighting their potential for orthodontic applications [28, 29].

This study aims to improve the mechanical properties of 3D-printed dental clear resin by enhancing the interfacial bond between the wire and resin. The surface morphology of modified wires and the interfacial shear strength were evaluated at the single-wire level. Tensile strength, elastic modulus, and stress relaxation of wire-embedded 3D-printed clear resin were measured. This innovative approach of integrating stainless steel wires into 3D-printed clear resin systems aims to provide a versatile solution with enhanced mechanical properties, expanding the range of orthodontic treatment options.

## Materials and methods

### Specimen Preparation

#### Preparation of wire sample and surface treatment

**Fig. 1:**
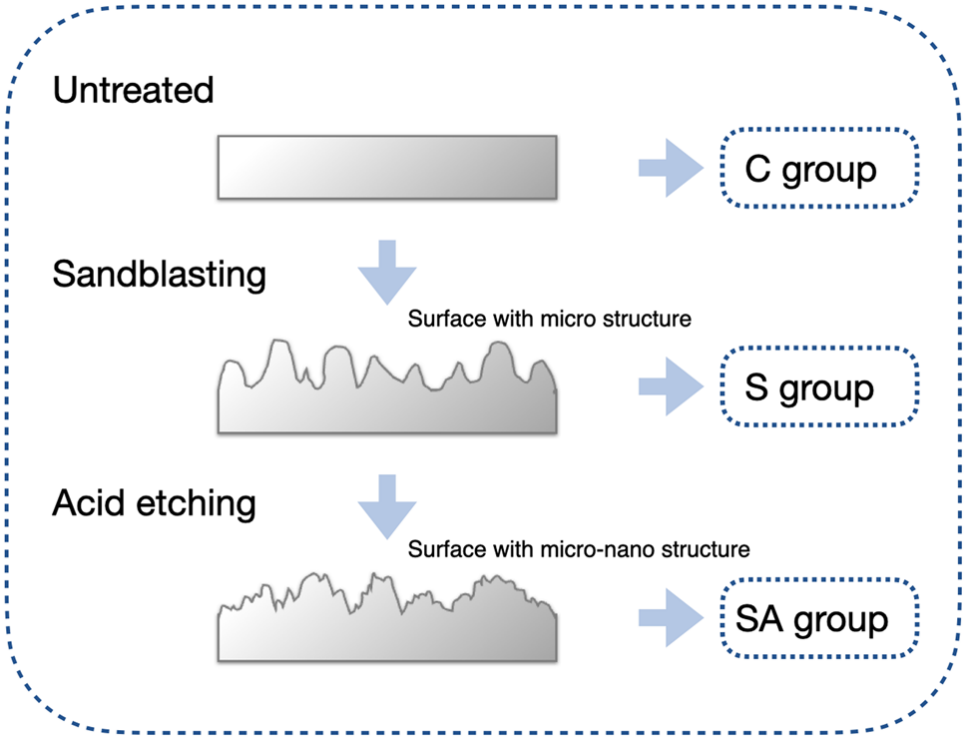
Schematic of the surface treatment on stainless steel wire

Stainless-steel wires (STSS1925, G&H Orthodontics, IN, USA) were divided into three groups based on surface treatments: control (C), sandblasting (S), sandblasting and acid etching (SA). Before surface treatments, all specimens were ultrasonically cleaned in acetone, ethanol, and deionized water for 10 min each, followed by air drying. The specific treatments and materials used are detailed in Tables 1 and 2, and a schematic illustration of the surface treatment process on the wire is shown in Fig. 1.

For groups S and SA, the wire surfaces were sandblasted using 50 μm aluminum oxide particles at a pressure of 3 bars and a distance of 10 mm for 60 s to achieve a uniform dull finish using a sandblaster (SandStorm Prestige I/O-801700, Vaniman, CA, USA). Following sandblasting and ultrasonic cleaning, wires in group SA were immersed in an acid solution (a 1:1 mixture of 10 wt% HCl and 10 wt% H_2_SO_4_) at 60 °C for 30 min, then ultrasonically cleaned with deionized water and air-dried at room temperature. Then, the silane coupling agent (SCA) primer (Monobond Plus, Ivoclar Vivadent, Schaan, Liechtenstein) was applied to the surface of the SA wires using a microbrush. The primer was allowed to react for 60 s to facilitate solvent evaporation, and then gently air-dried to remove the excess according to the manufacturer’s recommendations. The SCA enhances interfacial shear strength (IFSS) between the wire surface and clear resin through micro/nano mechanical interlocking and chemical bonds formed by the SCA infiltrating the wire’s surface pores [30]. In this study, SCA is applied to increase the chemical bond and provide bonding sites between the sandblasted and acid etching wire and clear resin. All specimens were stored at 23 °C / 50% relative humidity for 24 h before testing.

**Table 1:** Experimental stainless steel wire surface treatments

Group	Surface treatment protocols
C	No surface treatment
S	Sandblasting with 50 μm aluminum oxide at 3 bars for 60 s
SA	Sandblasting with 50 μm aluminum oxide at 3 bars for 60 s, etched with acid solution for 30 min at 60 °C, coating with SCA for 60 s

**Table 2:** General information of materials used in this study

Material	Commercial name, manufacturer	Description
Silane primer	Monobond Plus, Ivoclar Vivadent AG, Schaan, Liechtenstein	Alcohol solution of silane methacrylate, phosphonic acid acrylate, and sulphide methacrylate
Stainless steel wire	STSS 1925 14” Lengths, G&H Orthodontics, CA, USA	
Clear resin	Tera Harz TC*-*85 (DAC), Graphy, Seoul, Korea	

#### Preparation of pull-out test specimens

**Fig. 2:**
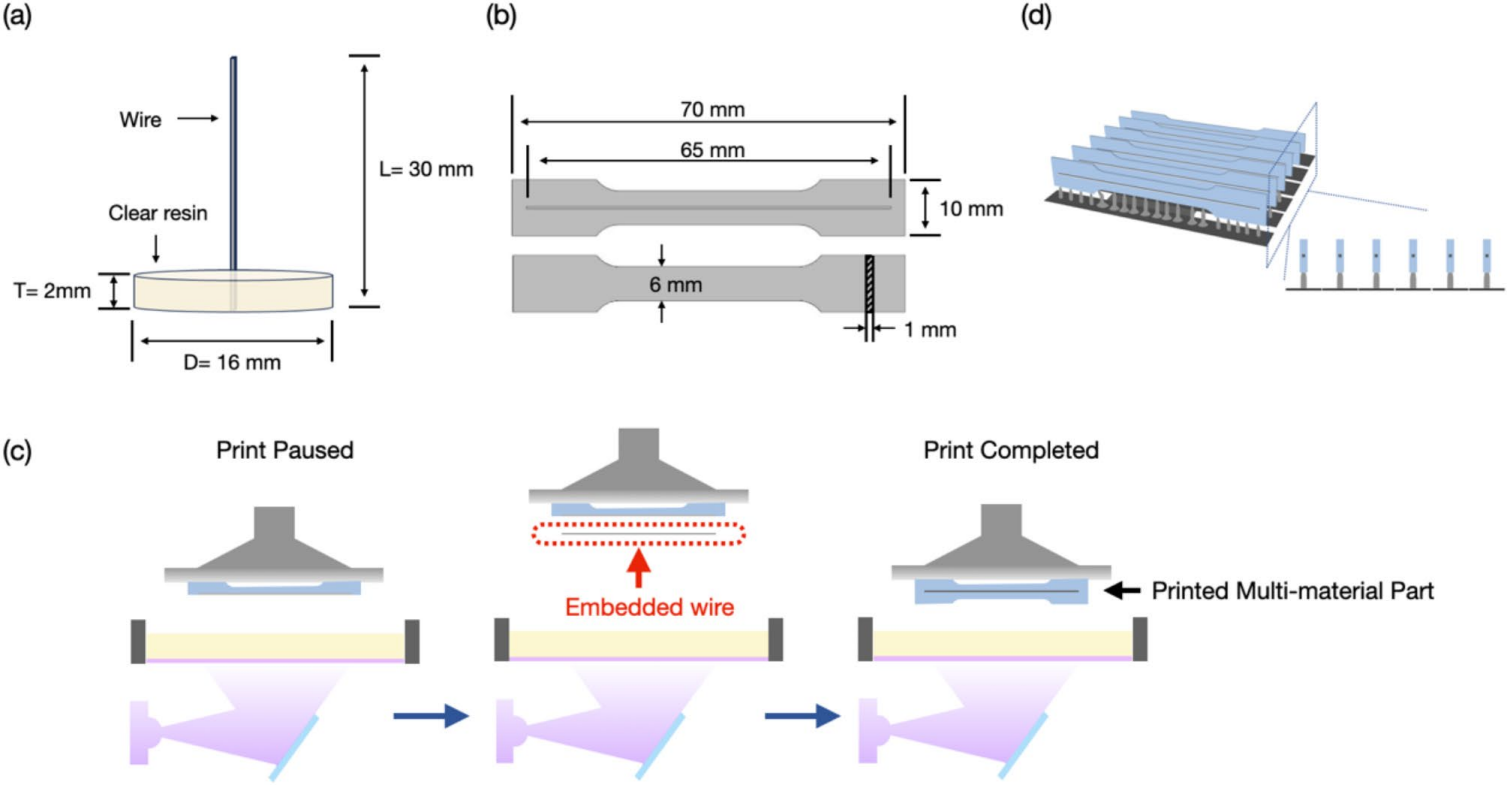
(**a**) Pull-out tests specimen dimensions. (**b**) 3D-printed specimen dimensions. (**c**) Multi-material 3D printing process used for clear resin embedded with wire. (**d**) Build orientation of 3D-printed specimens

In this research, a single-wire pull‐out test method was used to evaluate the interfacial shear strength (IFSS) of wire-resin adhesive specimens. The specimens were prepared as follows: wires with a length of 30 mm were subjected to three different surface treatments. The pull-out test specimens were fabricated using the setup illustrated in Fig. 2a. A polytetrafluoroethylene (PTFE) mould (D: 16.0 mm, T: 2.0 mm) was filled with the clear resin (Tera Harz TC-85; Graphy, Seoul, Korea) to prepare the disc-shaped pull‐out test specimens (*n* = 10 per group). The prepared specimens were cured for 60 min in the UV curing machine (ARUM Dentistry, Daejeon, Korea).

#### Design and 3D printing process

Dumbbell-shaped specimens for the tensile and stress relaxation tests were designed in standard tessellation language (STL) format using SolidWorks (Dassault Systèmes, France) in accordance with ISO 527-1:2019(E). The 3D-printed specimens were designed as dumbbell-shaped specimens (1 mm x 10 mm x 70 mm) and a rectangular cavity in the center of the specimens with dimensions of 0.46 mm in width, 0.76 mm in depth, and 65 mm in length. This cavity was designed to accommodate the embedded wire during the printing process. A schematic representation of the specimen design is shown in Fig. 2b.

Four groups of 3D-printed samples were prepared, as detailed in Table 3. To ensure consistency and minimize the influence of material thickness on the mechanical properties of the specimens, a uniform thickness of 1 mm was maintained for all samples [13]. The slicing process was performed using Chitubox v2.0 (Shenzhen CBD Technology Co., Shenzhen, China), and all specimens were printed using a Sonic Mini 4 K printer (Phrozen Technology, Hsinchu City, Taiwan). The specimens were printed with a layer thickness of 50 μm under 405 nm blue-violet light using liquid crystal display (LCD) technology. During the printing process, the printer was paused at the 206th layer, corresponding to the position of the rectangular cavity, to insert the wire into the cavity and then printing continued, as illustrated in Fig. 2c-d. After printing, the specimens were post-cured for 60 min in the post-curing machine (ARUM Dentistry, Daejeon, Korea).

**Table 3:** 3D-printed samples for four groups

Group	Description
P	3D-printed clear resin without wire
C	3D-printed clear resin with control wire
S	3D-printed clear resin with sandblasted wire
SA	3D-printed clear resin with sandblasting and acid-etching wire

### Surface characteristics of wires

To evaluate the surface characteristics of the wires, a scanning electron microscope (SEM) (SU 1510; Hitachi, Japan) was used to observe the surface morphology of each group at 15.0 kV with 2000 magnifications. The surface roughness and morphology of the wires were analyzed using atomic force microscopy (AFM) (Dimension Edge with ScanAsyst; Bruker Corporation, Billerica, USA). For each group (*n* = 6), the average surface roughness (R_a_) was measured and calculated.

### Static mechanical properties

#### Interfacial strength measurement (single-wire pull-out test)

The influence of surface modifications on interfacial strength was evaluated using a pull-out test model adapted from a previous study [31]. A total of 30 pull-out test specimens were fabricated and tested to evaluate the IFSS of the wire-resin specimen using the universal testing machine (ElectroPuls E3000; Instron, Norwood, USA). All pull‐out tests were conducted at a 5.0 mm/min crosshead speed. The IFSS (*τ*_i_) was calculated using the maximum debonding load obtained from the pull-out tests, as described by the following equation:

1 $$\:{\tau}_{i}=\frac{{F}_{max}}{{A}_{0}}$$

where *F*_*max*_ is the maximum debonding load measured from the experiments, *A*_*0*_ is the original adhesive area.

#### Tensile strength

Tensile tests were conducted on four groups of dumbbell-shaped specimens (*n* = 10) using the universal testing machine (ElectroPuls E3000; Instron, Norwood, USA). The tests were performed at a constant crosshead speed of 5 mm/min, with simultaneous monitoring of force and displacement. Stress-strain curves were analyzed to determine the tensile strength and elastic modulus for each group.

### Dynamic mechanical properties

#### Stress relaxation test

Stress relaxation refers to the reduction in load required to maintain a constant deflection over time [32]. The stress relaxation tests were carried out in accordance with ISO 6914. The stress relaxation test setup was also performed using the tensile method on the universal testing machine (ElectroPuls E3000; Instron, Norwood, USA). An initial deflection corresponding to 1% strain was applied within the first 5 s and maintained for 6 h, during which the load relaxation was monitored. Force values were recorded at 0.02 s intervals. The stress relaxation curves were drawn based on the residual stress. To ensure statistical validity, three specimens from each group were tested, allowing for comparative analysis of the curves and accurate evaluation of the material behavior. The residual stress at 60, 120, 180, 240, 300, and 360 min were selected and analyzed to compare the differences among the four groups.

#### Cyclic stress relaxation

Clear aligners and retainers undergo repetitive deflection and force decay during insertion and removal cycles. To simulate the daily three-times use of a clear aligner during one-week clinical therapy, a 21-cycle mode was set on the universal testing machine (ElectroPuls E3000; Instron, Norwood, USA). Each cycle consisted of stretching the specimen to 1% strain over 5 s, holding it constant for 10 min, removing the load over 1 min, and maintaining zero stress for 3 min. Three specimens from each of the four groups were tested, resulting in a total of 12 specimens.

### Statistical analysis

Statistical analysis was performed using SPSS software (version 28.0; IBM, Armonk, NY, USA). The statistical significance of the interfacial strength, tensile strength, tensile modulus and stress relaxation among these different groups was studied using one-way analysis of variance (ANOVA) and Tukey’s HSD post hoc multiple comparisons after testing the normality and homogeneity of variance. The level of significance was set to 0.05.

## Results

### Surface characterization and roughness of stainless-steel wire samples

**Fig. 3:**
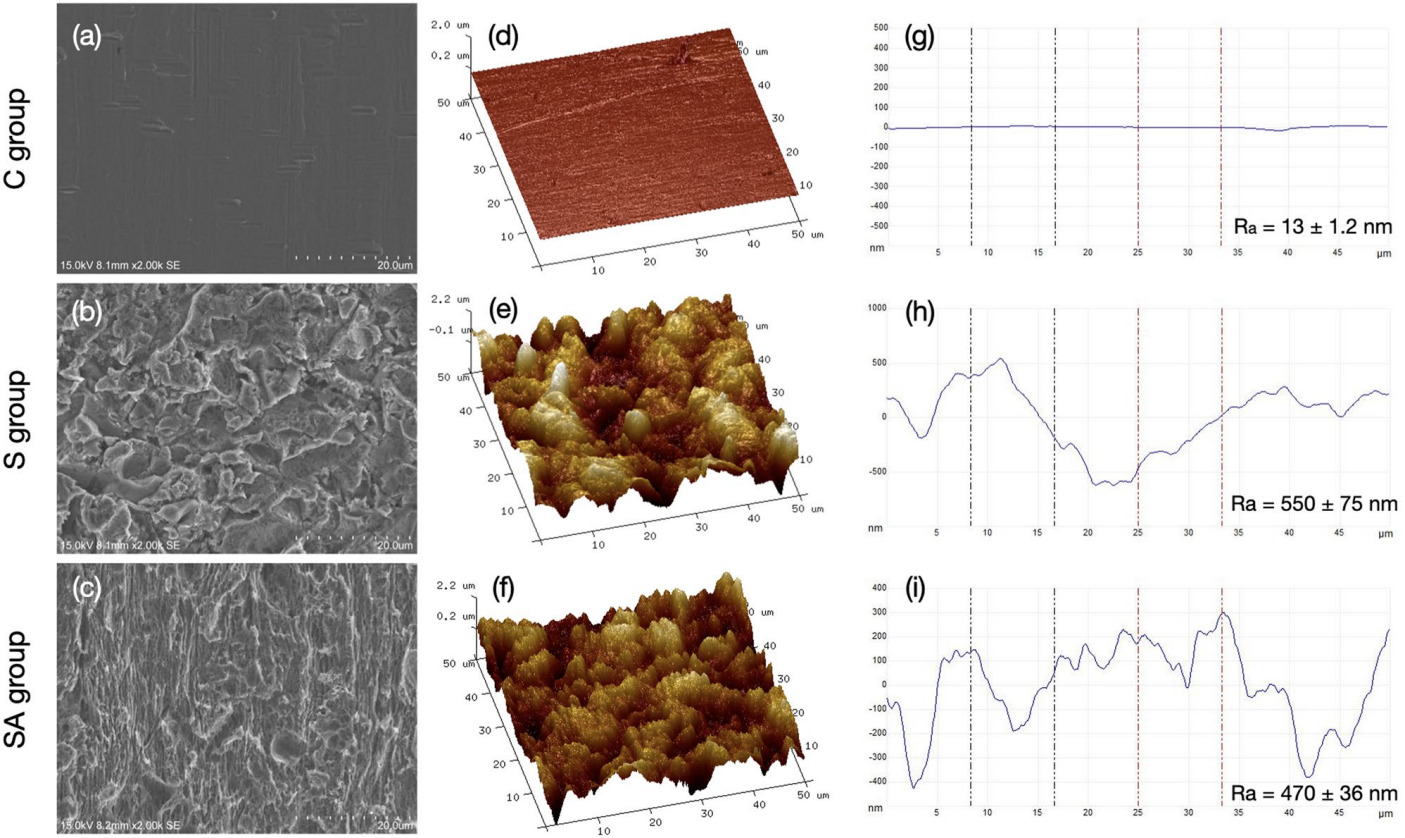
The representative microstructure of the stainless-steel samples prepared with different surface treatments. (**a**–**c**) Scanning electron microscopy images; (**d**–**f**) Amplitude atomic force microscopy images (50 × 50 µm^2^ scanning area); (**g**–**i**) Height line profiles with the average surface roughness

Figure 3 presents representative images of surface morphology for the different wire groups. The SEM images are depicted in Fig. 3a-c, the 3D AFM images are shown in Fig. 3d-f, and the topographical height profiles are extracted from the AFM images in Fig. 3g-i. The control group displayed a relatively smooth surface with slight scratches in Fig. 3a and d. The sandblasted group sample exhibited a coarse surface covered with irregular micro-scale craters, as shown in Fig. 3b and e. In contrast, the SA group showed irregular gaps and micropores as seen in Fig. 3c and f. This indicates that the SA group showed micro- and nano-scale grooves, pits, cavities, and coarser roughened morphologies compared to the S group. The combined sandblasting and acid-etching processes significantly increased the surface area and altered the surface morphologies, as evident in the groove topographies in Fig. 3g-i. The R_a_ values for the three different surface treatment groups are summarized in Fig. 3g-i. The R_a_ of the C group was 13 ± 1.2 nm. The surface roughness increased significantly to 550 ± 75 nm after sandblasting. After sandblasting and acid-etching, rough surfaces were obtained with the surface roughness of 470 ± 36 nm. Statistical analysis showed significant differences in surface roughness among the treatment groups (*P* < 0.001).

### Interfacial shear strength measurement (single-wire pull-out test)

**Fig. 4:**
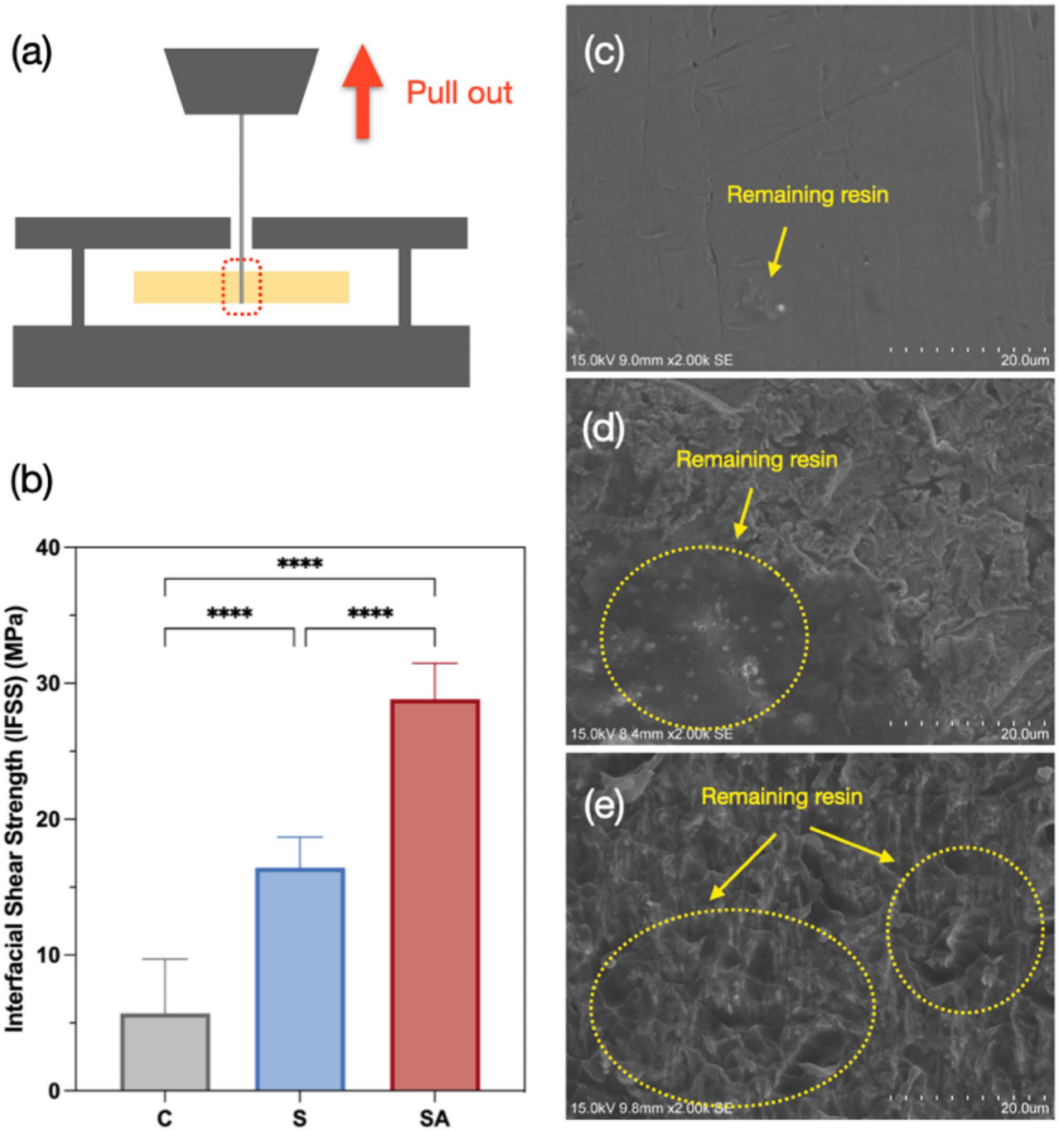
(**a**) The schematic illustration of the pull-out test; (**b**) the Interfacial shear strength value of different groups (**** *P* < 0.0001); (**c**–**e**) SEM images of the resin composites containing on the wire after the IFSS test: (**c**) C group, (**d**) S group, and (**e**) SA group

Figure 4a illustrates the schematic of the pull-out test, while Fig. 4b presents the IFSS results and statistical analysis. The interfacial shear strength of the control group is 5.7 ± 4.0 MPa. Sandblasting increased the IFSS by 2.8 times, raising it to 16.4 MPa. The sandblasting and acid-etching significantly enhance adhesion, resulting in a fivefold increase to 28.8 MPa. The morphologies of the fractured region were examined using SEM, as shown in Fig. 4c-e. The control group exhibited a smooth surface with minimal resin residue (Fig. 4c), indicating weak adhesion. The S group displayed partial resin retention on the wire surface in Fig. 4d. In contrast, the SA group demonstrated substantial resin residues within the micro-nano structure on the wire surface, as shown in Fig. 4e. This indicates a strong interfacial bond between the wire and the clear resin in the SA group, leading to fractures within the resin side rather than at the wire/resin interface. The calculated IFSS values further confirmed that the SA group exhibited superior performance compared to the control group. This improvement can be attributed to the increased surface roughness, which enhances the contact area, promotes mechanical interlocking between the wire and the clear resin, and facilitates stronger chemical bonding through the application of SCAs.

### Tensile strength and elastic modulus

**Fig. 5:**
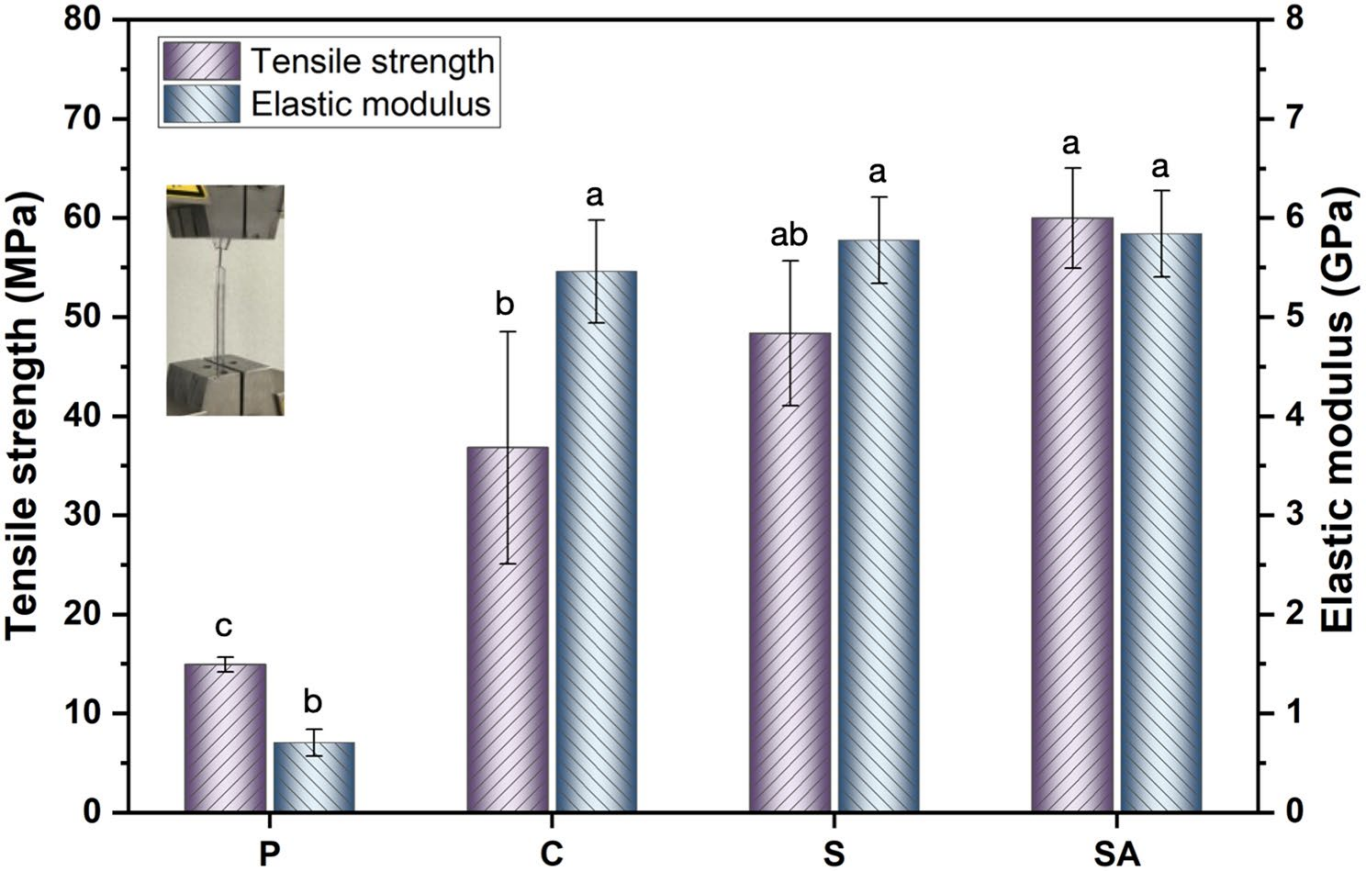
The static mechanical tensile strength and elastic modulus of different groups

The tensile strength and elastic modulus results are represented in Fig. 5. Embedding wires significantly enhanced the tensile strength and elastic modulus of the 3D-printed clear resin, with increases of two-fold and seven-fold, respectively. Specifically, the tensile strength increased from 14.9 MPa in the P group to 36.8 MPa in the C group. The SA group demonstrated the highest tensile strength at 60.0 MPa, approximately four times higher than the P group. However, there was no statistically significant difference between the tensile strength of the SA and S groups. Similarly, the elastic modulus was significantly higher in the wire-embedded groups compared to the pure resin group. The SA group exhibited an elastic modulus of 5.8 ± 0.4 GPa, while the P group showed the lowest value of 0.7 ± 0.1 GPa. The elastic modulus of the SA group was eight times higher than the P group. However, no significant differences were found among the C, S, and SA groups. The elastic modulus slightly improved from 5.5 GPa in the C group to 5.8 GPa in the SA group after sandblasting and acid-etching. These results indicate that wire embedding significantly improved the elastic modulus, but no statistically significant differences were observed among the different surface treatment groups.

### Stress relaxation test

**Fig. 6:**
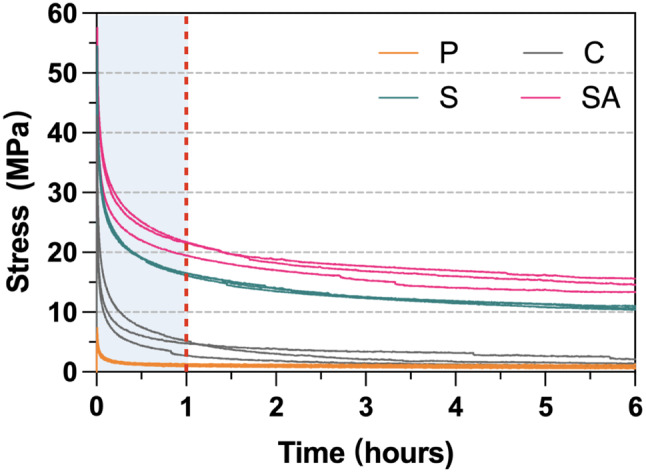
The stress-time curves of all groups tested over 6 h

The stress relaxation curves for the four groups over a 6-hour period under 1% total strain are shown in Fig. 6, with mean initial and residual stress values summarized in Table 4. All specimens exhibited rapid stress relaxation during the first hour, with the rate gradually leveling off from 3 to 6 h. The curves for each group were highly consistent, indicating excellent repeatability. Over the 6-hour observation period, the P group displayed the lowest initial and residual stress values, decreasing from 6.7 MPa to 0.9 MPa, representing the greatest stress decay. In contrast, the SA group exhibited the highest initial stress and residual stress at 14.5 MPa at the end of the test.

**Table 4:** Comparison of initial and residual stress of the different groups over 6 h

Group	Initial stress, MPa (SD)	Residual stress, MPa (SD)					
		60 mins	120 mins	180 mins	240 mins	300 mins	360 mins
P	6.7 (0.8) ^c^	1.1 (0.2) ^d^	1.0 (0.2) ^d^	1.0 (0.3) ^d^	0.9 (0.2) ^d^	0.9 (0.23) ^d^	0.9 (0.3) ^d^
C	36.7 (5.5) ^b^	4.3 (1.35) ^c^	2.9 (1.0) ^c^	2.3 (1.0) ^cd^	2.1 (1.0) ^cd^	1.8 (0.8) ^cd^	1.5 (0.5) ^cd^
S	53.7 (0.8) ^a^	16.4 (0.2) ^b^	13.8 (0.3) ^b^	12.4 (0.1) ^b^	11.7 (0.2) ^b^	11.2 (0.3) ^b^	10.7 (0.3) ^b^
SA	56.1 (2.7) ^a^	20.9 (1.2) ^a^	18.0 (0.9) ^a^	16.7 (1.2) ^a^	15.7 (1.5) ^a^	15.1 (1.3) ^a^	14.5 (1.2) ^a^

*Different lowercase superscript letters indicate statistically significant differences among groups (*P* < 0.05)

**Fig. 7:**
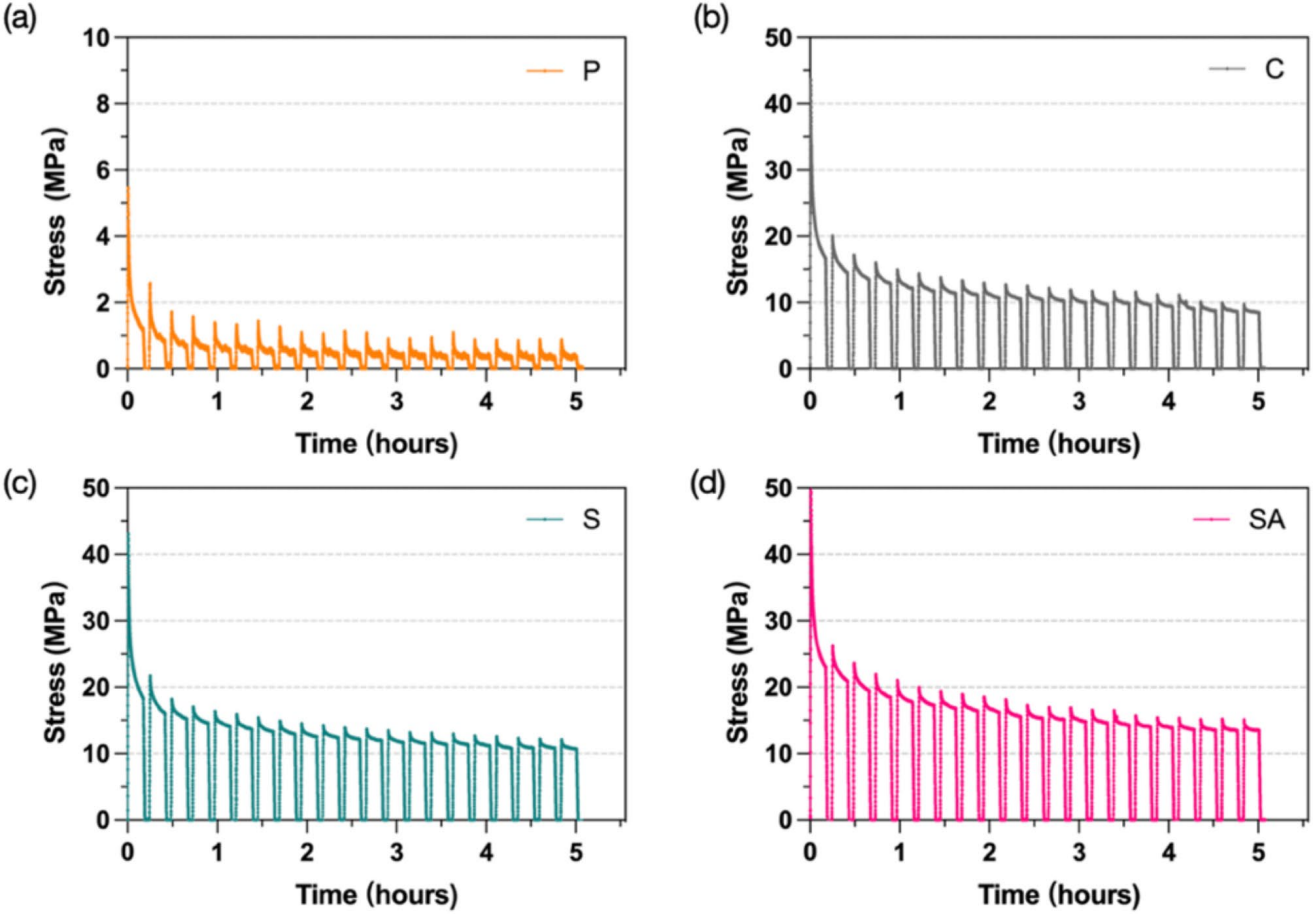
**Representative stress–time curves of all groups tested after 21 cycles.** (**a**) P group, (**b**) C group, (**c**) S group, (**d**) SA group

For cyclic stress relaxation, the residual stress decreased with repeated cycles across all four groups. Figure 7 illustrates the representative stress relaxation cycle curves for the four groups during the 21 cycles under 1% total strain, with mean initial and residual stress values detailed in Table 5. The SA group exhibited the highest initial stress at 21.4 MPa and the lowest percentage of stress decay. In contrast, the P group showed the lowest initial and final stress values, decreasing from 3.7 MPa to 0.6 MPa, indicating the greatest decay. The SA group also retained the highest percentage of residual stress, while the P and C groups displayed the lowest, as shown in Table 5. However, no significant difference was observed between the S and SA groups. The presence of embedded wire and surface treatment enhanced the stiffness of the clear resin samples and improved the adhesion between wire and resin.

**Table 5:** Comparison of initial and residual stress of the different groups after 21 cycles

Group	Initial stress, MPa	Residual stress, MPa	Residual stress, %
P	3.7 ± 0.4 ^b^	0.6 ± 0.1 ^c^	15.4^b^
C	49.8 ± 1.4 ^a^	8.3 ± 1.6 ^b^	16.6 ^b^
S	50.2 ± 2.1 ^a^	17.0 ± 1.8 ^a^	33.9 ^a^
SA	50.4 ± 1.9 ^a^	21.4 ± 1.1 ^a^	42.4 ^a^

*Different lowercase superscript letters indicate statistically significant differences among groups (*P* < 0.05)

## Discussion

This study introduces an innovative approach for integrating clear resin and stainless-steel wire using 3D printing technology while comparing the effects of different surface treatments to improve the adhesion between the resin and wire. The results demonstrate that the hybrid material exhibits enhanced mechanical properties due to the combination of clear resin and wire produced by hybrid additive manufacturing.

### Surface characteristic

The S group exhibited a surface roughness 50 times greater than the untreated C group. The sandblasting process generated irregular craters, valleys, and retained alumina particles, resulting in a coarse surface morphology. In contrast, the SA group displayed micro-scale pits and honeycomb structure micro-roughened morphology, consistent with the previous study [33]. The results indicated that both the S and SA groups exhibited increased surface roughness, although the SA group had lower roughness than the S group. Acid etching eliminates impurities but also blunts the sharp peaks and crests induced by sandblasting. This suggests that acid etching does not always increase roughness, a finding supported by Cao et al. [34]. Although the peaks and crests were blunted, honeycomb-shaped micro pits effectively increased the bonding area. Considering the passivation after acid-etching, the reduction in surface roughness seems justifiable. Surface roughness is mainly determined by sandblasting, as the holes from acid etching are only a few microns [35]. These findings indicate that the combined sandblasting and acid etching process can create a micro-nano structure with low roughness.

### Interfacial shear strength

The IFSS of stainless-steel wires with different surface treatments reflects the connection between surface microstructure and interfacial bonding. Pull-out tests were conducted to evaluate the micromechanical adhesion between the clear resin and the wires. As shown in Fig. 4b, the IFSS of the S group was three times higher than the C group, indicating that the sandblasting enhances IFSS by increasing surface roughness. The irregular craters and grooves generated by sandblasting increase the contact area between the wire and resin, thereby improving micromechanical interlocks [27]. Furthermore, the IFSS of the SA group was two times that of the S group. This improvement is attributed to the combined effects of acid etching and the application of SCAs on the wire surface. Acid etching increases the thickness of the bonding interface, while SCAs enhance both mechanical interlocking and chemical bonding. The formation of additional hydrogen bonds through SCAs further strengthens the chemical bonding at the interface [36]. Overall, sandblasting and acid etching significantly enhance interfacial shear strength, making it a recommended technique for improving the bond between composite materials and stainless-steel wires.

### Tensile strength

The tensile strength is a critical property for orthodontic clear appliances to withstand stress and long-term use [37]. This study evaluated the static mechanical properties of four groups of direct 3D-printed clear resin embedded with modified wire. The P group exhibited the lowest tensile strength, while the SA group demonstrated the highest strength due to enhanced mechanical interlocking and chemical bonding resulting from surface treatments. The tensile strength of thermoplastic aligner materials ranges from 35 MPa to 78 MPa [38–40]. In comparison, the 3D-printed clear resin (Tera Harz TC-85) displayed lower tensile strength than other 3D-printed clear resin materials [41, 42] and thermoplastic aligner materials [17, 43, 44]. Thus, it is necessary to improve the mechanical properties of 3D-printed clear resins by utilizing multiple materials. The tensile strength was effectively enhanced by embedding wires and applying surface treatments to the metal wires.

### Elastic modulus

The elastic modulus is another key factor in determining the force exerted by orthodontic appliances and their ability to effectively move and maintain tooth positions throughout treatment [45]. There is a correlation between the exerted force and elastic modulus of the material [46]. In this study, the elastic modulus of the P group was 0.7 GPa, significantly lower than other 3D-printed clear resin [42, 47] and thermoplastic aligner materials [38–40, 48], which typically range from 1.3 GPa to 2.9 GPa. Previous studies have suggested that direct 3D-printed clear appliances may exhibit inferior mechanical properties compared to thermoformed materials, potentially limiting their effectiveness in tooth movement [20]. Kohda et al. [46] reported that an elastic modulus of approximately 2.8 GPa is optimal for effective tooth adjustments. In this study, embedding wire increased the elastic modulus of the C group to 5.5 GPa compared to the P group, demonstrating that wire integration effectively enhances elastic modulus [49]. However, no significant differences in elastic modulus were observed among SA, S, and C groups, indicating that surface treatments do not significantly impact this property [50]. For clear aligner treatment, a lower elastic modulus is preferable in the initial stages for patient comfort, while aligners with embedded wire can provide increased elastic modulus and strength during the middle and final stages of treatment. This integration method enhances the mechanical properties of 3D-printed appliances without requiring complex post-processing, offering a practical solution for various orthodontic applications.

### Stress relaxation

Clear aligners are subjected to prolonged wear and repeatedly inserted in the oral cavity, leading to force decay over time. This study investigated stress relaxation and cyclic stress relaxation to simulate the long-term performance and repeated use of different groups. All tested groups exhibited rapid stress decay within the first few hours under constant strain, followed by a plateau phase, which is typical behavior for polymers and consistent with previous studies [51, 52]. The P group had the lowest residual stress at 0.9 MPa, indicating difficulty maintaining sufficient force for effective tooth movement. Similar findings have been reported in prior research, with initial forces showing exponential decay over time [53]. Additionally, Cremonini et al. observed that 3D-printed Tera Hartz TC-85 exhibited stress relaxation properties comparable to the P group in this study, with decay percentages ranging from 90 to 100% [54]. Clear aligner materials typically have an elastic modulus approximately 40 to 50 times lower than that of conventional Ni-Ti archwire [45], indicating that aligner materials can deform significantly with little force. Clinically, dental crowding can be resolved with a single archwire, whereas the same condition requires multiple aligners. The residual stress levels in thermoplastic materials typically range between 38% and 82% [55]. Shirey et al. reported that the residual stress levels in other 3D-printed aligner materials were significantly lower than those of thermoformed samples after 2 h at 2% strain [20]. In this study, the C group showed the residual stress of only 1.5 MPa after wire embedding, likely due to insufficient bonding between the resin and wire. In contrast, the S and SA groups demonstrated significant improvements in stress relaxation compared to the P and C groups, indicating that surface treatments enhance the material bonding and improve mechanical properties.

To simulate daily insertion and removal of aligners, a cyclic stress relaxation test was conducted to represent one week of clinical use [41]. As cycles were repeated, stress relaxation increased and residual stress decreased, mirroring the trends observed over 6 h. The stress decay induced by repeated aligner insertion is expected to be reduced after embedded wire and surface treatment. The P group exhibited an initial static stress of 3.7 MPa, which rapidly decreased to 0.6 MPa after 21 cycles. A related study reported similar stress relaxation behavior in 3D-printed clear resin Tera Harz TC-85, with a residual static force reaching 1 N [16]. Significant stress relaxation may reduce the predictability of appliance performance over time. Although wire embedding without surface treatment increased residual stress, the C group still exhibited considerable stress relaxation similar to the P group. The SA group achieved the highest residual stress at 42.4% after 21 cycles, demonstrating that wire embedding and surface treatments can reduce force decay under cyclic loading.

**Fig. 8:**
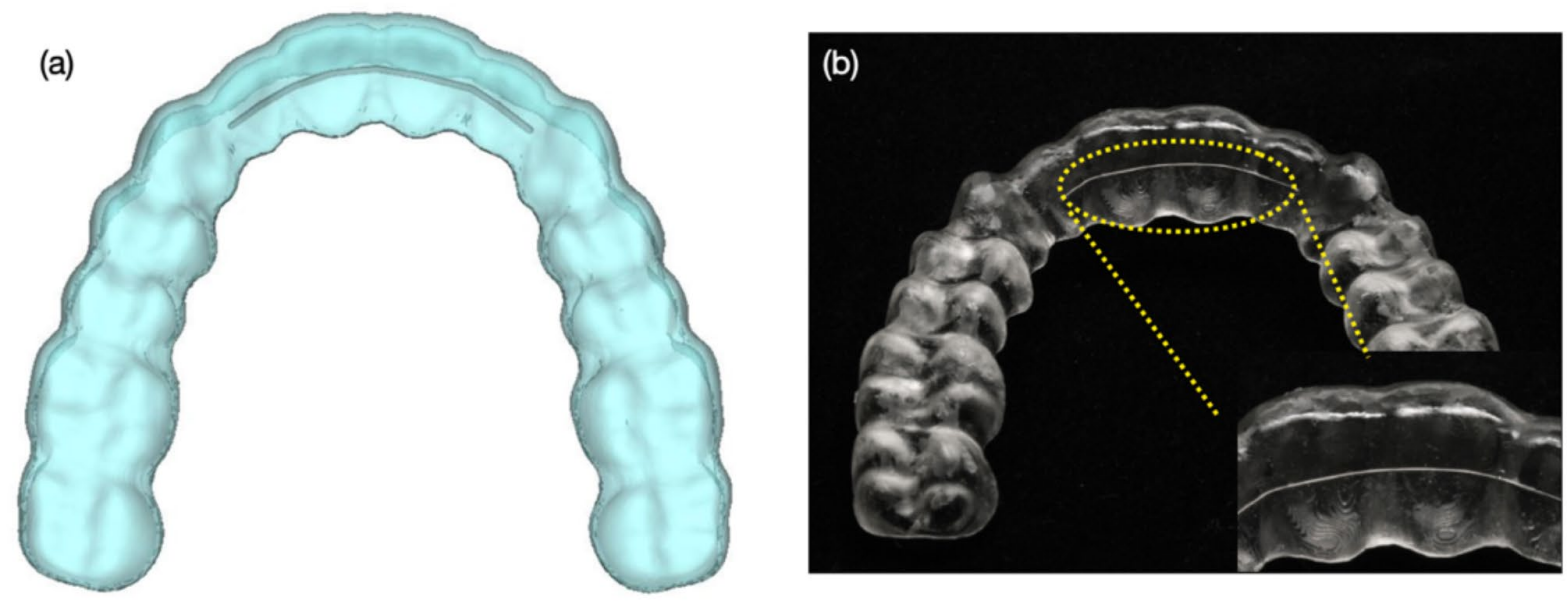
(**a**) the virtually designed clear aligner with wire. (**b**) the final 3D-printed clear aligner with embedded wire

This study presents a novel manufacturing concept for 3D-printed clear resin, enabling customizable metal reinforcements based on specific clinical needs. The newly designed multi-material 3D-printed clear appliance integrates the mechanical strength of embedded wire with the aesthetic and elastic properties of resin, potentially overcoming the limitations of conventional orthodontic devices as shown in Fig. 8. The limitation of this study is its in vitro design, highlighting the need for further experiments and clinical case studies to validate its clinical applicability. Future research should consider intraoral conditions, virtual simulations, and in vivo studies to provide more comprehensive information. This study provides foundational insights into the characteristics of 3D-printed multi-material orthodontic appliances. This innovative technique can be widely applied not only for orthodontic clear aligners and retainers but also for other removable dental appliances such as splints, expanders, mandibular advancement devices, and night guards.

## Conclusion

The conclusions of this study are summarized as follows:

1. The static and dynamic mechanical properties of 3D-printed clear resin are significantly enhanced by embedding orthodontic wire treated with sandblasting and acid etching (SA).
2. Stainless steel wires with micro-nano roughened surfaces exhibit a significantly increase in interfacial shear strength, with sandblasting and acid etching treatment yielding the highest IFSS values.
3. The tensile strength of the 3D-printed clear resin increased significantly by four times, and the elastic modulus improved by seven times embedding the SA group wire. However, the surface treatment of the wire does not affect the elastic modulus.
4. The residual stress in the P and C groups is nearly ten times lower than that in the S and SA groups after 6 h at 1% strain. The SA group exhibits the highest residual stress and lowest stress decay after 21 cycles.

## Data Availability

The data that support the findings of this study are available from the corresponding author and the first author upon reasonable request.
